# Responsible Use of Artificial Intelligence to Improve Kidney Care: A Statement from the American Society of Nephrology

**DOI:** 10.1681/ASN.0000000929

**Published:** 2025-11-07

**Authors:** Navdeep Tangri, Wisit Cheungpasitporn, Stanley D. Crittenden, Alessia Fornoni, Carmen A. Peralta, Karandeep Singh, Len A. Usvyat, Amy D. Waterman

**Affiliations:** 1 Department of Internal Medicine, University of Manitoba, Winnipeg, Manitoba, Canada; 2 Mayo Clinic, Rochester, Minnesota; 3 Quantum Health, Redondo Beach, California; 4 Department of Medicine, University of Miami, Miami, Florida; 5 Habitat Health, San Mateo, California; 6 Joan and Irwin Jacobs Center for Health Innovation, University of California, San Diego, San Diego, California; 7 Renal Research Institute, New York, New York; 8 Houston Methodist Hospital, Houston, Texas

**Keywords:** kidney, kidney disease, kidney failure, nephrology, artificial intelligence

## Abstract

Artificial intelligence (AI) is rapidly transforming the delivery of kidney care through predictive analytics, machine learning, deep learning, and generative AI technologies. To meet this challenge, the American Society of Nephrology convened an AI Workgroup to provide a framework for the responsible use of AI in nephrology. The group outlines foundational principles to guide AI development: prioritizing patient benefit, ensuring clinician oversight, and advancing innovation in high-burden disease areas. Its set of foundational assumptions are grounded in the physician always being in the loop and an overarching goal to benefit patients with kidney diseases. This review provides an overview of the clinical uses of AI in nephrology and offers practical guidance for nephrologists seeking to incorporate AI into CKD and AKI management, dialysis, and transplantation care. It also highlights key challenges—such as data quality, equity, transparency, and clinical integration—that must be addressed to ensure the responsible and effective implementation of AI in kidney care.

## Introduction

Artificial intelligence (AI) refers to computational systems designed to emulate human cognitive functions, such as learning, reasoning, and decision making. Nephrology provides an ideal environment for AI applications because of the abundance of data generated during routine care. Dialysis sessions, laboratory results, and electronic health records (EHRs) produce structured and longitudinal datasets, whereas nontraditional data sources, such as environmental, behavioral, and socioeconomic factors, can further enhance AI insights.^1^ With ongoing advancements in computational capabilities, algorithm design, and data integration, AI can be incorporated with traditional patient care to improve outcomes and better address the complexities of kidney disease management.^1,2^ However, there is also a need for human oversight and ethical use of these tools. The US Food and Drug Administration plays a critical role in guiding the development and implementation of AI technologies in health care.^3^ Its digital health and AI glossary defines terms such as adaptive learning referring to systems that evolve on the basis of new data and explainability, which emphasizes the need for AI models to be understandable by clinicians. These concepts are pivotal for ensuring that AI systems align with established medical practices, uphold patient safety, and support clinical decision making.

**Figure 1:**
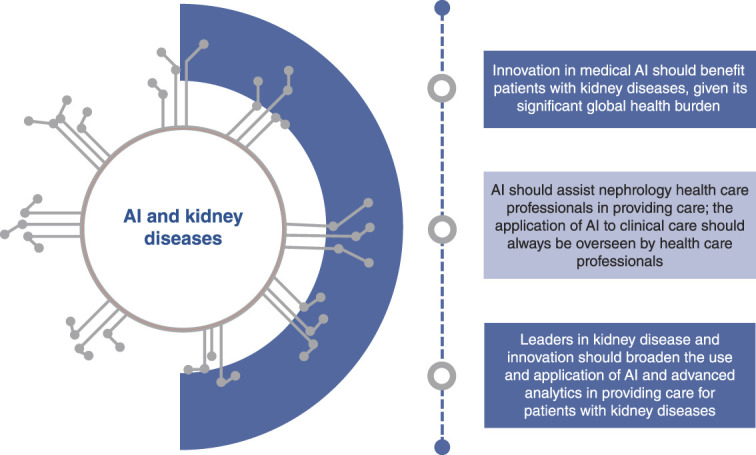
**Guiding principles of the American Society of Nephrology Workgroup on responsible AI and kidney diseases.** AI, artificial intelligence.

There is also a fundamental role for nephrologists to play in guiding the ethical and effective use of AI in our specialty. Recognizing this, the American Society of Nephrology formed an AI Workgroup to develop a framework for responsible AI use in nephrology and to educate nephrologists about the opportunities and challenges associated with AI. The AI Workgroup has two overarching goals: (*1*) to encourage the development of AI solutions to benefit patients with kidney diseases and (*2*) to promote a framework for development of AI that is clinically oriented and ensures that the physician remains continuously involved (Figure 1).

The following review outlines the opportunities and potential pitfalls of AI use in nephrology, establishes a framework for ethical and effective use, and positions nephrologists to leverage these powerful new tools to optimize patient care.

## Applications of AI in CKD

AI is poised to revolutionize kidney care by enabling more precise, personalized, and efficient approaches to treatment. The use of AI/machine learning (ML) may help integrate and automate the workflow, leading to better detection and management of CKD.

Despite more than 2 decades of evidence, particularly in patients with diabetes, screening rates for CKD remain low in nearly every country.^4^ Although clinical practice guidelines and underlying evidence strongly endorse a dual marker/heatmap approach to diagnosis, staging, and prognostication in patients with CKD, current clinical practice continues to be largely eGFR based, leaving significant gaps in appropriate testing, diagnosis, and treatment.^5^

**Figure 2:**
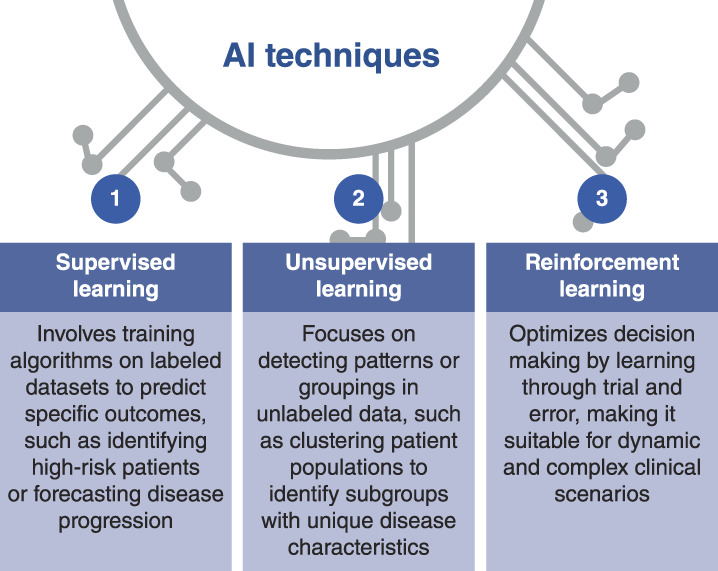
Pillars of machine learning.

Subfields within AI include ML and its subset, deep learning (DL). ML, also referred to here as predictive AI, uses data-driven algorithms to identify patterns and make predictions, whereas DL uses artificial neural networks to process data in sophisticated ways, mimicking human cognition.^1^ These approaches have shown promise in identifying clinical patterns, predicting adverse events, and guiding treatment decisions across various medical specialties, including nephrology (Figure 2).^1^ Recent developments in DL/image processing using AI provide meaningful alternatives to our current screening paradigms. Imaging-based software used in home testing kits (Minuteful Test) can detect the albuminuria signal from a smartphone, a dipstick, and a home collection kit and can provide a semiquantitative estimate of albuminuria.^6^ In addition, retinal-based imaging using a point-of-care device can accurately identify CKD without laboratory tests in the field.^7^ These AI applications are particularly useful in screening individuals outside of traditional health care settings.

The availability of treatments to slow or reverse CKD has made it imperative to recognize and treat the disease early, particularly for patients at a higher risk of progression to kidney failure. In later stages of CKD, equations such as the kidney failure risk equation can provide accurate discrimination and calibration and inform decisions about nephrology referral, vascular access planning, and kidney transplantation.^8^ However, most patients with CKD have a preserved or relatively preserved eGFR.

Recently, investigators have developed CKD progression models using ML methods and routinely collected or highly specific (biomarker-based) laboratory data. The Klinrisk model, developed using international laboratory data repositories and validated in more than 5.8 million individuals, has been shown to accurately predict CKD progression using only a complete blood count, metabolic panel, and urine albumin-creatinine ratio (UACR) with an area under the curve of 0.8–0.88.^9,10^ Renalytix AI developed an approach using three proprietary biomarkers and clinical data to provide early prediction of diabetic kidney disease with an area under the curve of 0.74–0.77.^11^ These models complement the kidney failure risk equation and can predict progression at earlier stages, enabling earlier treatment.

Approaches to enhance CKD diagnosis and risk prediction must seamlessly fit into clinical workflows and promote optimal management. The TrajVis tool implemented at Wake Forest is one example of integrating an AI-driven clinical decision support system into electronic medical records or laboratory information systems to provide clear decision aids or order sets that link laboratory tests to diagnosis/risk stratification and guideline-recommended treatments.^12^ Test reports from both the Klinrisk and KidneyIntelx results also provide guideline-based decision support when a high-risk patient is identified. These models are being used in clinical care and have been shown to improve appropriate testing of UACR and prescription of guideline-directed medical therapy.

## Challenges to Implementation

There has been a lack of progress in UACR testing in the past 20 years because of clinical inertia and the persistence of an eGFR-centered view of CKD diagnosis and management. Without a truly connected EHR and innovations in the application of AI-based clinical decision support systems, progress in this area is likely to be slow. We believe that rigorous implementation and evaluation of existing models and efforts to improve clinical workflow and integration must be prioritized. Growing acceptance of AI solutions in enterprise EHRs should allow for more seamless integration of existing tools, which must be evaluated and improved. These tools also need to be tested in both primary care and nephrology clinics where patients' clinical needs may differ.

## Applying AI to AKI

AKI diagnosis relies on detecting a rise in serum creatinine, which occurs hours to days after tubular damage, resulting in delayed and often ineffective interventions. Regression-based models can predict AKI when there is a discrete event such as cardiopulmonary bypass, but AI-based tools may have advantages in more heterogeneous settings.

By analyzing real-time patient data—such as trends in serum creatinine, urine output, vital signs, and comorbidities—these models can effectively identify at-risk patients with greater accuracy than regression-based methods.^13–15^

Integrating AI into EHRs may also enable real-time monitoring and dynamic risk stratification for patients with AKI.^11^ It may also support proactive management by recommending evidence-based interventions, such as fluid adjustments and nephrotoxic medication modifications, allowing clinicians to focus on critical decision making rather than manual data interpretation.

AI-integrated EHRs may also provide advanced trend visualizations and predictive models of kidney function. The success of such systems requires seamless integration with existing EHR platforms, robust real-time data pipelines, and collaboration between clinicians and AI developers to ensure user-friendly interfaces.

## Applying AI to Critical Care Nephrology

AI has the potential to support the optimal timing of dialysis initiation in the intensive care unit (ICU) by evaluating clinical parameters such as fluid balance, electrolyte levels, and hemodynamic stability.^15–17^ Reinforcement learning models could be particularly effective in tailoring dialysis prescriptions to individual patient needs, potentially minimizing complications such as intradialytic hypotension.^17,18^

**Figure 3:**
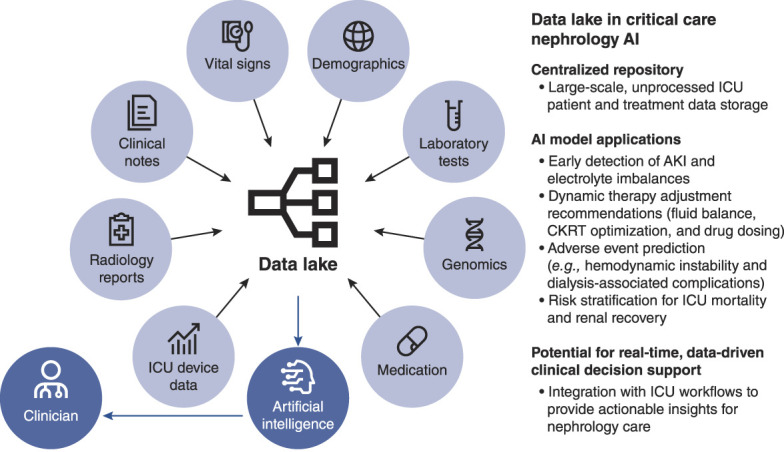
**ICU data lake for critical care nephrology.** CKRT, continuous kidney replacement therapy; ICU, intensive care unit.

The proposed concept of an ICU data lake has the potential to significantly enhance the application of AI in KRT decision making (Figure 3). By centralizing real-time and historical data from diverse ICU sources, the ICU data lake could create a unified repository for comprehensive patient monitoring. This repository may enable longitudinal tracking of patient status and support dynamic, predictive analytics tailored specifically to critical care nephrology. AI algorithms applied to this data lake could predict adverse events, such as AKI onset or electrolyte imbalances, and optimize continuous KRT prescriptions by dynamically adjusting parameters on the basis of patient-specific thresholds. Notwithstanding, it is important to note that improvement in prediction does not necessarily mean improvement in clinical care, and outcomes should be measurable and evaluated using scientific approaches.^19,20^

## Challenges in Implementation

Critical care environments generate vast amounts of data, yet the quality, consistency, and completeness of these data vary widely. Using AI to improve nephrology care/KRT initiation requires improving data quality, integrating data sources within EHRs, and updating predictive models as clinical guidelines change. Issues such as missing values, measurement errors, and inconsistent documentation within EHRs often hinder AI model training and deployment.^15,19,20^

Current development of AI models in critical care nephrology often uses retrospective data from single institutions, which may not reflect the diversity of patient demographics, clinical practices, or health care environments.^19–21^ In addition, models on the basis of physiologic data cannot account for physician preferences or resource constraints regarding KRT initiation in the ICU. This raises concerns about the generalizability of AI models, particularly in underserved populations or regions with varying health care resources. Performance drift, defined as a loss of discrimination or calibration over time, can also occur because of inadequate validation and poor generalizability.^22,23^

We believe that AI models require routine updates incorporating clinical guidelines, new data, and changing patient populations. Similarly, robust quality control processes are essential to ensure consistent performance and to detect potential errors. Developing standardized protocols for model maintenance and validation will be critical for addressing these issues and preserving long-term model reliability and trust in clinical workflows.

## Applying AI in Dialysis Care

The highly standardized and data-rich nature of dialysis therapy makes it a prime candidate to benefit from AI. Each dialysis session generates extensive longitudinal patient-level data, encompassing treatment parameters, biosignals, and patient demographics, which are systematically captured in EHRs and other digital systems.^23–25^ These large datasets provide an ideal foundation for the development and application of AI models designed to improve patient outcomes and operational efficiency.

AI has already demonstrated its utility in dialysis through predictive modeling, treatment optimization, and decision support. For example, the anemia control model, a certified AI-driven decision support system, helps optimize the management of anemia in patients on hemodialysis by recommending precise doses of erythropoietin-stimulating agents. This model significantly improved hemoglobin target achievement rates and reduced medication use, leading to cost savings and better patient outcomes.^26,27^

AI-based prediction models for hospitalization and mortality have been used for over a decade.^24^ AI has also advanced the prediction of intradialytic hypotension, a common complication during dialysis.^28^ DL models that integrate predialysis features and historical session data have achieved high predictive accuracy, enabling clinicians to make preemptive adjustments to treatment protocols.^24^

Recent advancements in AI have enabled the use of diverse data modalities in dialysis care, extending beyond traditional numeric datasets. For example, analysis of ultrasounds or other imaging data using AI may identify vascular access complications, such as aneurysms.^29^ Similarly, AI might be used for real-time monitoring of treatment conditions by listening to audio signals, such as those generated by dialysis machines, or for monitoring patients between sessions using wearable biosensors.^30^

Large language models (LLMs) excel in processing and interpreting complex, unstructured data, such as clinical notes, patient histories, and educational materials. For instance, LLMs can assist health care professionals in summarizing large volumes of patient data, generating personalized treatment recommendations, or even providing real-time decision support during dialysis sessions.^31^

Moreover, LLMs can enhance patient engagement and education. Chatbot applications powered by LLMs have already been piloted to deliver tailored health information and improve self-care for patients on peritoneal dialysis.^32^ These tools can also break language barriers by generating multilingual content for diverse patient populations, thereby improving accessibility and equity in care delivery.

## Challenges in Implementation of AI in Dialysis Care

Despite these advancements, several challenges hinder the broader adoption of AI in dialysis. Collection and integration of socioeconomic determinants of health remains inadequate.^2,24,25^ Data collected outside the clinic are not yet fully integrated into in-center hemodialysis workflows.

The high costs of developing, validating, and maintaining AI models, coupled with regulatory hurdles, deter widespread adoption. Transparency issues related to the black-box nature of AI algorithms further complicate implementation because they may erode trust among clinicians and patients.^24,25^ Addressing AI challenges related to data integration, socioeconomic factors, and economic incentives will be critical to paving the way for more personalized, efficient, and equitable dialysis care.

## Applying AI in Transplantation

The use of AI in kidney transplantation has the potential to improve outcomes across the transplant continuum through predictive modeling, treatment optimization, and education and decision support. It could help expand access to living donor kidney transplantation, optimize deceased donor organ allocation, and improve post-transplantation outcomes.

## Boosting Transplant Rates and Living Donation

Kidney transplant is the optimal treatment for patients with kidney failure. Yet, disparities in access for Black, Hispanic, and other historically underserved and socioeconomically disadvantaged subgroups have been well documented.^33–35^ ML-driven risk indices have been developed using demographic, clinical, and socioeconomic data to identify patients at risk of failing to complete transplant evaluation, waitlisting, or transplantation, enabling targeted interventions to reduce disparities and improve access.^36^ ML approaches can outperform traditional risk prediction models by handling complex, nonlinear, and large-scale data, offering superior accuracy, adaptability, and integration of different variables.^37,38^

Living donation increases kidney availability and is associated with better survival and quality of life compared with other KRTs.^39–41^ Although national attitudes toward living donor kidney transplantation are positive, there remains a gap between the number of patients on the waiting list and the number of living donors.^39–42^ Leveraging AI/ML to data collected through social media and other digital platforms can assess public sentiment, behavioral cues linked to donation, and barriers around donation. It may offer personalized opportunities for engagement and education of those likely to become donors or answer questions about becoming a donor.^43,44^

## Optimizing Deceased Donor Organ Matching, Utilization, and Allocation

The kidney donor risk index predicts graft failure risk on the basis of donor characteristics, and the estimated posttransplant survival (EPTS) score combines recipient factors to predict post-transplant survival; both are integrated into the Kidney Allocation System to prioritize high-quality kidneys for recipients with lower EPTS scores.^45–48^

Despite these prediction tools, high-risk kidneys remain prone to discard, especially those with discordant expanded criteria donor and kidney donor profile index (KDPI) indicators.^46,47^ Several studies have used AI algorithms to improve donor–recipient matching by incorporating more variables such as blood type, tissue compatibility, medical history, and geographic distribution, offering personalized indices for kidney allocation through tools such as the KDPI-EPTS online model.^47,49–52^ Studies using ML algorithms show that even high-KDPI organs result in improved survival and measurable benefits for patients with a high risk of death on dialysis in regions with prolonged waiting times.^51^ Although these advancements are promising, AI/ML adoption remains limited to pilot programs and research, with clinical integration still in progress.

## Improving Post-transplant Outcomes

The primary strength of using ML models lies in their capacity to process complex, high-dimensional data. However, their advantage over traditional prediction models for post-transplant outcomes remains controversial.^52–54^ As an example, the iBox tool, which uses a transparent logistic regression model integrating demographic, functional, immunologic, and histopathologic variables, has been extensively validated for predictive modeling across international cohorts. The European Medicines Agency accepts it as a surrogate end point for graft failure in clinical trials, demonstrating the level of robustness, interpretability, and regulatory alignment that AI/ML models must achieve to support clinical and trial decision making.^55^

AI/ML-based approaches have shown significant potential in optimizing immunosuppressive therapy for kidney transplant recipients. AI-based dosing systems have demonstrated high accuracy in predicting drug levels and dosages, minimizing biases and improving treatment precision.^56–61^ In addition, ML models incorporating genetic polymorphisms and patient demographics have enhanced the prediction of tacrolimus bioavailability and post-transplant diabetes risk.^57,62–64^

## Challenges of AI in Transplantation

In kidney transplantation, the availability of data needed for AI modeling and its complexity and the possibility for bias pose significant barriers. Patient datasets are often smaller and more fragmented compared with areas such as CKD where large, standardized datasets are available. Moreover, kidney transplantation relies heavily on integrating diverse data types—donor/recipient characteristics, immunologic and histopathologic data, genetic profiles, and longitudinal outcomes—requiring complex processing and robust algorithms.

AI/ML in kidney transplantation often struggles with poor generalizability because of population-specific data from smaller cohorts. Models must account for regional, socioeconomic, and racial and ethnic differences in patient populations, making generalization more complex.

The use of AI/ML in kidney transplantation raises significant ethical concerns, particularly around fairness and equity in organ allocation. There is a risk of exacerbating disparities if interventions disproportionately focus on patients deemed most likely to succeed in getting transplanted or maintaining a transplant, rather than addressing the needs of those at highest risk. Ensuring fairness and transparency in prioritizing patients is critical, necessitating careful ethical consideration when applying risk indices to clinical decisions.

## Addressing Nephrology Education Challenges with AI

Currently, there is great variability in teaching and training approaches across nephrology fellowship programs. Although advances in medical education have been made, traditional methods such as didactic lectures, clinical rotations, and case-based discussions have limitations. These approaches may inconsistently teach standards, lack personalization, and fail to integrate emerging technologies such as AI. These failures may affect trainee readiness to use AI tools such as scribes, patient messaging applications, and clinical decision support in practice.

**Figure 4:**
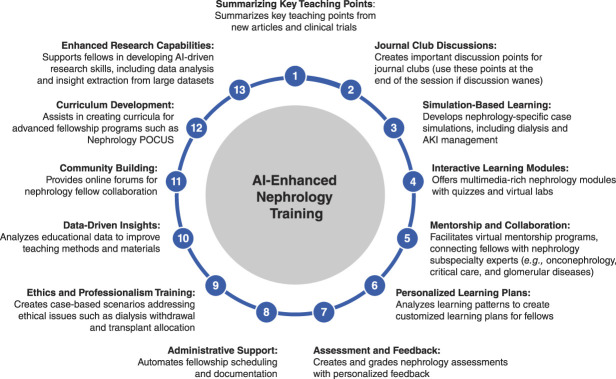
**The potential role of AI in enhancing nephrology fellowship training.** POCUS, point-of-care ultrasound.

AI may help integrate diverse educational content, clinical scenarios, and real-time data to enhance training precision and support personalized learning strategies.^65^ It has the potential to significantly improve fellowship training and prepare nephrologists for future challenges (Figure 4).^66,67^

AI tools have the potential to assist nephrology educators by summarizing key points from recent nephrology research articles and clinical trials with review and contextualization by attending physicians to ensure relevance and accuracy.^66^ These summaries can be used for lectures, fellow handouts, and study materials, providing learners with concise, up-to-date knowledge.

AI tools can also create important discussion points for journal clubs. These AI-generated points can be particularly useful at the end of journal club sessions, especially if there is a lull in the conversation, to help spark deeper discussions and analysis. However, attending nephrologists must continue to play a key role in confirming the validity of these discussion points and guiding the conversation toward meaningful clinical insights.

## Simulations and Personalized Learning Plans

Virtual reality offers an immersive, hands-on environment to explore kidney anatomy, observe disease progression, and simulate procedures such as dialysis setup, patient assessments, and post-transplant care in a risk-free setting.^68^ These approaches, supervised by attending physicians, ensure that fellows receive the critical feedback necessary for refining their diagnostic reasoning and clinical skills.

AI can also analyze learning patterns to create customized learning plans for nephrology fellows. These plans can cater to each fellow's individual strengths and weaknesses, ensuring a more targeted and effective educational experience. Nephrology educators can oversee these plans to ensure alignment with educational goals and clinical requirements.

## Challenges to Anticipate, including Plagiarism or Academic Dishonesty

AI tools pose significant risks to academic integrity because they can instantly generate polished content that fellows might use without attribution.^69^ Fellows risk becoming overly dependent on AI's convenience, potentially compromising their critical thinking and analytical skills.^70^ This dependency can reduce independent problem-solving abilities and lead to a superficial understanding of complex topics.

**Figure 5:**
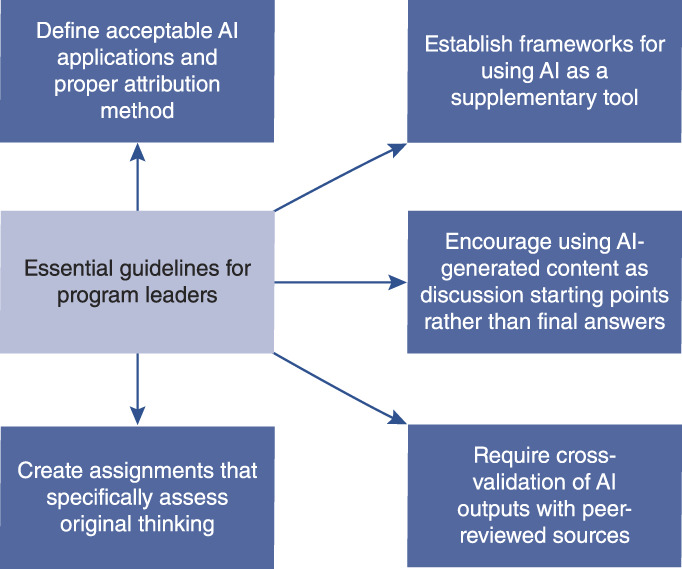
Structured guidelines for program leaders in medical education.

Institutions should implement clear guidelines that define acceptable AI use and establish proper attribution methods. Effective strategies include integrating AI awareness into academic integrity training, implementing AI-detection tools, and clarifying the distinction between legitimate uses (brainstorming and overcoming writer's block) and inappropriate applications (verbatim copying) (Figure 5).

Success lies in fostering responsible AI use while preserving the essential human elements of academic work—creativity, critical thinking, and ethical judgment. AI should enhance, not replace, the intellectual growth process.^70^

## Improving Efficiency and Reducing Administrative Burden

Generative AI's growing ability to extract, summarize, paraphrase, and interpret information in unstructured text has the potential to also improve clinician well-being and health system efficiency.^71^

AI is already being widely adopted to generate EHR documentation. The shift to electronic billing systems and the need for rigorous documentation to satisfy insurers' payment requirements and monitor quality metrics accelerated the use of this technology.^72^

Dictation systems and human scribes have been deployed to reduce clinicians' documentation burden. Scribes require ongoing training and coaching, exacerbated by high turnover in the position. AI scribing systems promise to overcome these limitations by transforming the audio of the visit into a clinical note within minutes using predefined clinician templates. These systems have produced mixed results in early studies, which shows that they reduce clinicians' cognitive burden but save only a few minutes per day.^73–76^

The number of electronic messages exchanged between patients and clinicians has continued to grow since the pandemic, making it an area ripe for innovation. In April 2023, Epic Systems announced a pilot of GenAI for drafting replies to patient messages.^77^ This pilot has since expanded to include broad rollouts at many health systems around the country, where evidence suggests that it may reduce cognitive burden but not necessarily yield substantial time savings.

GenAI also has shown potential to assist clinicians in drafting persuasive prior authorization letters or to appeal denials of prior authorization. Payors are also alleged to be using GenAI to create denial letters, which has prompted legislation to curb this use.^78^

There are also many potential back-office uses of AI in health care, which may include billing and quality measurements. GenAI tools may use clinical documentation to suggest billing codes or existing billing codes to suggest improvements in clinical documentation. Early evidence also suggests that automation of quality measurement through GenAI is possible, although real-world uses remain sparse.

## Challenges: Outcomes, Cost, Labor, Privacy, and Legal Uncertainties

The return on investment of implementing GenAI tools to reduce administrative burden can be viewed as a mix of efficiency gains, productivity gains, greater clinician well-being, and reduced clinician turnover. The limited effect on clinician time saving and the cost of the technologies may give health systems pause, but decreasing costs over time may make them more affordable and improve the return on investment. Consideration should be given to the potential adverse effect on labor demand arising from new innovations and service automation. The roles of administrative personnel who both support physicians and provide human contact with service users may be in competition with AI tools as technology improves. In addition, there is potential for de-skilling of health care professionals after exposure to AI tools, particularly for new health care professionals who may not have yet fully developed their clinical approach.^79^ The use of these technologies in direct patient care and patient data means that it is more important than ever to ensure their accuracy and that patients' privacy is protected. Enterprise contracts often come with privacy guarantees that go beyond what is available directly to consumers, including guarantees that data will not be used in model training. However, patients also deserve clear communication about the use of these technologies, their benefits, and risks. Finally, there remain uncertainties in the legality of language models that have been trained using copyrighted works.

## Summary

AI is transforming kidney care through predictive and generative innovations—from AKI and dialysis to transplantation and education. Although this review focused on the use of AI in certain areas of nephrology, its applications remain broad and can be applicable to other areas such as fluids and electrolyte disorders, hypertension, kidney stones, and glomerular diseases. By addressing barriers such as data quality, ethical concerns, integration complexity, and clinician trust, the field of nephrology can use AI to deliver more precise, equitable, and efficient care. Used responsibly, AI has the power to enhance outcomes for patients and the professionals who care for them.
